# Whole-Genome Sequencing-Based Characterization of a *Listeria monocytogenes* Strain from an Aborted Water Buffalo in Southern Italy

**DOI:** 10.3390/microorganisms9091875

**Published:** 2021-09-03

**Authors:** Rubina Paradiso, Marita Georgia Riccardi, Bianca Cecere, Nunzia Riccone, Roberto Scialla, Antonietta Anzalone, Anna Cerrone, Esterina De Carlo, Giorgia Borriello, Giovanna Fusco

**Affiliations:** 1 Istituto Zooprofilattico Sperimentale del Mezzogiorno, Via Salute 2, 80055 Portici, Italy; rubina.paradiso@izsmportici.it (R.P.); maritageorgia.riccardi@izsmportici.it (M.G.R.); bianca.cecere@izsmportici.it (B.C.); nunzia.riccone@cert.izsmportici.it (N.R.); roberto.scialla@izsmportici.it (R.S.); antonietta.anzalone@izsmportici.it (A.A.); anna.cerrone@cert.izsmportici.it (A.C.); giovanna.fusco@izsmportici.it (G.F.); 2 Centro di Referenza Nazionale Sull’igiene e le Tecnologie Dell’allevamento e delle Produzioni Bufaline, Istituto Zooprofilattico Sperimentale del Mezzogiorno, Via Salute 2, 80055 Portici, Italy; esterina.decarlo@cert.izsmportici.it

**Keywords:** *Listeria monocytogenes*, whole genome sequencing, water buffalo, virulence genes, antibiotic resistance

## Abstract

*Listeria monocytogenes* is a Gram-positive pathogen causing life-threatening infections both in humans and animals. In livestock farms, it can persist for a long time and primarily causes uterine infections and encephalitis in farmed animals. Whole genome sequencing (WGS) is currently becoming the best method for molecular typing of this pathogen due to its high discriminatory power and efficiency of characterization. This study describes the WGS-based characterization of an *L. monocytogenes* strain from an aborted water buffalo fetus in southern Italy. The strain under study was classified as molecular serogroup IVb, phylogenetic lineage I, MLST sequence type 6, Clonal Complex 6, and cgMLST type CT3331, sublineage 6. Molecular analysis indicated the presence of 61 virulence genes and 4 antibiotic resistance genes. Phylogenetic analysis, including all the publicly available European *L. monocytogenes* serogroup IVb isolates, indicated that our strain clusterized with all the other CC6 strains and that different CCs were variably distributed within countries and isolation sources. This study contributes to the current understanding of the genetic diversity of *L. monocytogenes* from animal sources and highlights how the WGS strategy can provide insights into the pathogenic potential of this microorganism, acting as an important tool for epidemiological studies.

## 1. Introduction

*Listeria monocytogenes* is a facultative intracellular pathogen ubiquitously present in natural environments with the ability to cause life-threatening infections both in humans and animals [1]. In ruminants, *L. monocytogenes* primarily causes uterine infections and encephalitis. Uterine infections induce late-term abortions or septicemia in neonates, while central nervous system (CNS) infection, mainly rhombencephalitis, is the most common clinical manifestation in animals and is associated with a high mortality rate [2,3]. In addition, *L. monocytogenes* can cause eye infection and keratitis in ruminants. Listeriosis may occur as a large outbreak within a farm, even though it most commonly affects a single or a few animals [4]. Indeed, infected animals are generally asymptomatic carriers but can spread this pathogen by fecal shedding, thus disseminating the pathogen in the farm environment [5,6].

Although listeriosis is quite a rare disease compared to other food- and feed-borne infections, it is associated with an extremely high fatality rate [3]. For this reason, its surveillance and control are a major public health concern. *L. monocytogenes* is characterized by a high genetic diversity and includes numerous strains, which are diversified into four evolutionary lineages with possible inter-strain differences related to virulence and transmission [3,6]. Serotyping was the first method developed for the discrimination of *L. monocytogenes* isolates, but it has low discriminatory power. Indeed, while this technique allows for the identification of thirteen serotypes, the vast majority of human and animal infections are caused by three serotypes only: 1/2a, 1/2b, and 4b [7,8,9]. A variety of other methods has been developed to characterize *L. monocytogenes* isolates, including EcoRI ribotyping, multilocus genotyping (MLGT), multilocus variable number of tandem repeats (MLVA), pulsed-filed gel electrophoresis (PFGE), multilocus sequence typing (MLST), and whole genome sequencing (WGS). At present, MLST and WGS, used for core genome MLST (cgMLST), are the most suitable methods for *Listeria* characterization in consideration of its largely clonal reproductive nature and high genetic diversity. Epidemiological tracking for surveillance and outbreak investigation requires high-resolution methods that are able to provide more accurate discrimination of the isolated strains. Moreover, the possibility of identifying the presence of virulence genes and correlating them with the reported disease severity would be highly desirable in order to, in the future, predict the strains’ virulence according to specific virulence markers.

The aim of this study was, therefore, to perform a WGS-based characterization of an *L. monocytogenes* strain isolated from the lungs of an aborted water buffalo fetus in southern Italy to gather more information on strain-specific virulence.

## 2. Materials and Methods

### 2.1. L. monocytogenes Isolation, DNA Isolation, Library Preparation, and Sequencing

The *L. monocytogenes* strain characterized in this study was isolated from a water buffalo fetus delivered from an asymptomatic mother and consigned to the Istituto Zooprofilattico Sperimentale del Mezzogiorno for complete necropsy examination, as previously reported by Esposito and colleagues [10]. *L. monocytogenes* isolation from liver, abosum content, and lung samples was carried out as described by the World Organization for Animal Health (OIE) in the Manual of Diagnostic Tests and Vaccines for Terrestrial Animals [11]. *Listeria* colonies were selected according to biochemical traits such as Gram-positive, catalase-positive, oxidase-negative, and beta-hemolytic on Blood Agar (Oxoid, Heysham, UK), and identified by miniaturized biochemical procedures with Vitek2-compact (bioMérieux, Marcy-l’Étoile, France).

Pure *L. monocytogens* colonies isolated from lungs were grown on Ottaviani and Agosti agar (ALOA agar) (Oxoid) at 37 °C for 24 h and collected for DNA extraction by using the QIAamp DNA mini kit (Qiagen, Hilden, Germany) following the manufacturer’s instructions. The DNA concentration was evaluated using a Qubit 3 (ThermoFisher Scientific, Waltham, MA, USA). For whole genome sequencing, approximately 100 ng of genomic DNA was used. A DNA library with fragments of 400 bp in length was prepared using the Ion Xpress Fragment Library kit (Life Technologies, Carlsbad, CA, USA) according to the manufacturer’s instructions. The DNA was sequenced on an Ion Gene Studio S5 platform (ThermoFisher Scientific, Waltham, MA, USA) at approximately 25X coverage. The generated sequencing reads were checked using FastQC, and low-quality sequences were removed by using PRINSEQ lite. High-quality sequences were successfully assembled using SPAdes [12] version 3.15.0. The bacterial genome was assembled in 22 contigs for a total length of 2,876,538 bp, with a mean contig length of 130,752 nucleotides (maximum contig length: 645,034 nucleotides, and minimum contig length: 328 nucleotides) and an N50 contig value of 3, with an N50 length value of 295,773. Contigs were aligned with the reference sequence NZ_CP010346_1 using ABACAS version 1.3.1. The draft assembly of the sequenced strain was deposited in the *Listeria* Pasteur MLST database (https://bigsdb.pasteur.fr/listeria, last access on 28 April 2021) under the accession ID 77784.

### 2.2. WGS-Based Characterization of L. monoytogenes

WGS data were used for the molecular characterization of the *L. monocytogenes* strain under study by identifying the phylogenetic lineage, the PCR serogroup, the MLST and cgMLST type, the Clonal Complex (CC), and the presence of virulence and antibiotic resistance genes with the bioinformatic tools available on the BIGSdb-Lm platform (https://bigsdb.pasteur.fr/listeria, last access on 28 April 2021). A minimum spanning tree was constructed based on the genes used for MLST classification (*abcZ*, *bglA*, *cat*, *dapE*, *dat*, *ldh*, and *lhkA*) by using the software GrapeTree on a list of WGS isolates chosen from the Institute Pasteur database, including all publicly available *L. monocytogenes* serogroup IVb isolates from Europe.

## 3. Results

The *Listeria monocytogenes* strain under study was isolated from the lungs of an aborted water buffalo fetus exhibiting severe abdominal and moderate pleural serohematic effusions, with the presence of mild pericardial serohematic fluid [10].

Molecular typing indicated that this strain belonged to molecular serogroup IVb, phylogenetic lineage 1 (Table 1). This strain was assigned to a seven-loci MLST sequence type 6 (ST6) and Clonal Complex 6 (CC6). Further classification using cgMLST classified it into CT3331 sublineage 6 (SL6).

Screening of WGS data for 17 antimicrobial resistance genes indicated the presence of four genes (Table 1): *fosX* (fosfomycin), *lmo0919*(*lin*) (antibiotic ABC transporter ATP-binding protein), *norB* (multidrug efflux pump), and *sul* (dihydropteroate synthases). Acquired antibiotic resistance traits were not detected.

Screening for 92 virulence genes revealed the presence of 61 genes (Table 1), including most of the major *Listeria* virulence factors, such as those involved in bacterial cell wall modification (*gtcA*), adherence to host cells (*inlJ*, *fbpA*, and *lap*), actin-based motility (*actA*), invasion (*inlA* and *inlB*), intracellular growth (*lplA1* and *prsA2*) and bile resistance (*bsh*), immune evasion (*pdgA* and *oatA*) and immune modulation (*inlC*, *inlK*, and *intA*), and exoenzyme production (*plcA*, *mpl*, and *plcB*). Finally, the strain under study displayed the presence of not only pathogenicity island LIPI-1 (*prfA*, *plcA*, *hly*, *mpl*, *actA*, and *plcB* genes) and internalin *inlABCEGHIJKP* markers, which are widely distributed in each *L. monocytogenes* serotype, but also LIPI-3 (*IlsAGYDP* genes), which is classically associated with *Listeria* serotype IVb (Table 1).

**Figure 1:**
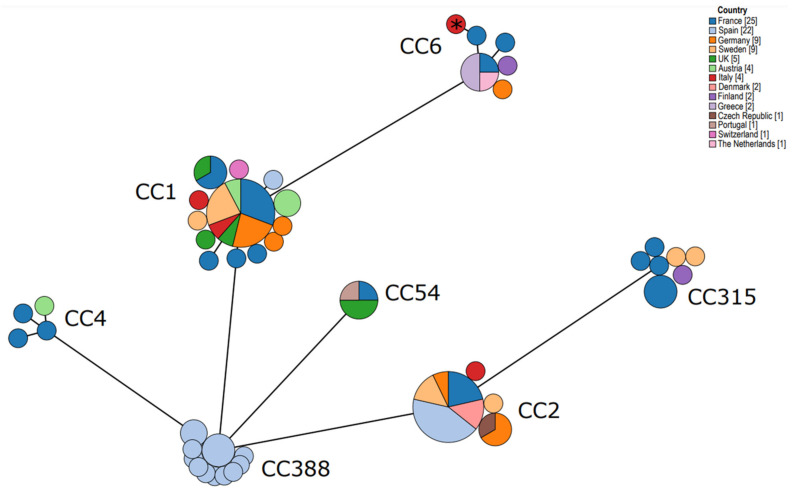
Minimum spanning tree constructed based on the gene scheme used for MLST characterization from a list of 88 sequenced isolates (including the strain under study) chosen from the Institute Pasteur database by selecting all the available *L. monocytogenes* serogroup IVb isolates from Europe. Clusterization is displayed by CC and country of origin. Asterisk indicates the *L. monocytogenes* strain under study.

A minimum spanning tree was constructed based on the gene scheme used for MLST characterization from a list of 88 sequenced isolates (including the strain under study) chosen from the Institute Pasteur database by selecting all the publicly available *L. monocytogenes* serogroup IVb isolates collected from Europe in order to highlight the genomic differences among strains (Figure 1). All the isolates could be divided into seven distinct clusters, corresponding to CC1, CC2, CC4, CC6, CC54. CC315, and CC388, respectively.

The strain under study clusterized with the other eight CC6 isolates, three of which were from France, two from Greece, one from Germany, one from the Netherlands, and one from Finland. The French CC6 isolates exhibited different cgMLST types: CT458, CT481, and CT467, respectively. Unfortunately, the information on the cgMLST type of the other isolates in the CC6 cluster was missing. The source of origin of the different isolates included in the analysis is reported in Table 2. The CC54 cluster included only isolates of human origin and the CC388 cluster included only isolates of animal origin (15 isolates from Spanish deer and wild boars), while the other CC clusters exhibited more variability in the source of origin (Table 2).

## 4. Discussion

This study describes the WGS-based characterization of an *L. monocytogenes* strain isolated from the lungs of an aborted water buffalo fetus in southern Italy. This strain exhibited a molecular serogroup IVb. This serotype is among those most frequently found in association with clinical cases both in animals and humans [7,8,9]. Indeed, this serotype has been found to replicate in murine monocytes/macrophages more efficiently than other *Listeria* serotypes, supporting the evidence of higher pathogenicity of serotype 4b strains, with particular reference to a greater ability of dissemination through the host body [13].

Our strain was classified as belonging to lineage I, ST6, CC6. Another strain, ST6, has been isolated and sequenced from a fatal listeriosis case in an adult man in Italy [14], even though ST6 type is not among the most represented *L. monocytogenes* ST types in Italy [15]. The clonal complex CC6, together with CC37, has been found to be statistically significantly associated with abortion in ruminants [2]. CC6 strains were connected with both human and animal infections as well as food, raw meat, and food processing environment contamination [3,16]. Moreover, in humans, the clones CC1, CC2, CC4, and CC6 of lineage I have been described as hypervirulent pathogens that are responsible for severe clinical diseases [17]. Indeed, these CCs mainly include clinical isolates with a low frequency of isolates from food and the environment [18]. This trait might be due to the presence, in these CCs, of many specific virulence genes with a low frequency of genes promoting survival under different environmental stress conditions [17,19]. According to this assumption, WGS-based characterization of the strain under study revealed the presence of a conspicuous number of virulence genes. Indeed, our *L. monocytogenes* strain harbored not only the LIPI-1 (a six-gene cluster) and several internalin genes, all widely distributed in each *L. monocytogenes* serotype [20,21], but also LIPI-3 (an eight-gene cluster, coding for cytotoxic and hemolytic factors that have been shown to contribute to the virulence of *L. monocytogenes*) [22]. The association of LIPI-3 (Listeria Pathogenicity Island 3) and LIPI-1 (Listeria Pathogenicity Island 1) is considered to be responsible for the increased virulence in some *L. monocytogenes* strains [1]. In addition to these pathogenicity islands, our strain also harbored most of the major listerial virulence genes, including those involved in bacterial adherence to the host intestinal epithelium and survival at a low availability of oxygen and nutrients (*lap* and *fbpA*), the secretion of active protein products (*PrsA2*), immune system modulation and evasion (*pdgA*, *oatA*,and *intA*), the oxidation of macrophages, and the development of infection (*orfX*) [1]. Moreover, we found a specific gene cassette (3,071 bp) that consists of two genes, *gltA* and *gltB*, involved in the expression of teichoic acid-associated surface antigens in serotypes 4b-4d-4e [23]. In *L. monocytogenes* serotype 4b, *gtcA* and *gltA* have been implicated in serotype-specific glycosylation of the teichoic acid of the cell wall with galactose and glucose. The gene *gltA*, harbored by *L. monocytogenes* of the serotype 4b complex (serotype 4b and the closely related serotypes 4d and 4e) but no other serotypes, has been previously shown to be necessary for the placement of glucose on the teichoic acid of serotype 4b *L. monocytogenes* [23], while the gene *gtcA*, specific to serotype 4b, has been associated with the addition of adequate amounts of galactose and glucose to the teichoic acid of the listerial cell wall [24]. A number of other virulence genes have been found in the gene pool of the *L. monocytogenes* strain under study, confirming its role in the reported clinical abortion and demonstrating that ruminants are exposed to hypervirulent CCs. After all, ruminants have been shown to play an important part in the amplification and spread of *L. monocytogenes* in agricultural environments, with farmyards acting as natural reservoirs [25]. The presence of this pathogen in livestock farms poses a serious risk to both animal and human health due to possible contamination of the food and feed chain.

The minimum spanning tree constructed with publicly available European *L. monocytogenes* serogroup IVb isolates indicated that our strain clusterized with all the other CC6 isolates and displayed a wide distribution of CCs within countries and isolation sources. These results are consistent with the results reporting that the major clonal complexes implicated in clinical cases of listeriosis, that is, CC1, CC2, CC4, and CC6, are ubiquitous and characterized by a regional heterogeneity larger than surmised [16].

Finally, the presence of antimicrobial resistance genes was investigated in the strain under study. Antimicrobial resistance is recognized as a global public health threat and multidrug-resistant pathogens have been found in people, animals, foods, and the environment, including water, soil, and air [26]. Since the first detection of an antimicrobial-resistant *L. monocytogenes* strain in France in 1988 [27], many other antibiotic-resistant strains have been found [25,28,29]. Our strain displayed the presence of four intrinsic resistance genes, present in all *L. monocytogenes* isolates: *fosX* (fosfomycin), *lmo0919lin* (antibiotic ABC transporter ATP-binding protein), *norB* (multidrug efflux pump), and *sul* (dihydropteroate synthase), likely indicating possible resistance to fosfomycin (an analogous of phosphoenolpyruvate PEP, which inhibits bacterial cell wall biogenesis), lincomycin (a member of the lincosamide class, interfering with protein synthesis), quinolones (interfering with DNA replication), and sulfonamide (inhibiting folic acid synthesis), respectively.

## 5. Conclusions

The accuracy of genomic characterization provided by WGS analysis proves to be extremely useful for outbreak identification as well as epidemiological tracking systems. It also highlights the usefulness of WGS characterization in order to identify the pathogenic potential of bacterial strains in relation to the harbored virulence and antibiotic resistance genes. According to our knowledge, this study is the first report on WGS analysis of *L. monocytogenes* from water buffalo. Our results highlight the pathogenic potential of *L. monocytogenes* strains from domestic ruminants. These data can provide insight into the genetic diversity of this microorganism and help epidemiological investigations connected to this food- and feed-borne pathogen.

## Figures and Tables

**Table 1:** WGS-based characterization of the *L. monocytogenes* strain under study.

Molecular Characterization	Molecular Results
Taxonomy	Molecular serogroup IVb; phylogenetic lineage 1; ST6; CT3331; SL6; CC6
Antimicrobial resistance	*fosX, lmo0919(lin), norB, sul*
Major virulence genes	*gtcA, inlJ, fbpA, lap, actA, inlA, inlB, lplA1, prsA2, bsh, pdgA, oatA, inlC, inlK, intA, plcA, mpl, plcB*
*Listeria* pathogenicity islands	LIPI-1 LIPI-3
Other virulence genes	*gltA, gltB, prfA*, *hly*, *orfX*, *dltA*, *aut*_IVb, *iap*, *lpeA*, *vip*, *hpt*, *purQ*, *svpA*, *agrA*, *agrC*, *cheA*, *cheY*, *fur*, *lisK*, *lisR*, *stp*, *virR*, *virS*, *lgt*, *lspA*, *srtA*, *srtB*, *mdrM*, *comK*, *codY*, *pdeE*, LMON_RS01340, LMON_RS01345

**Table 2:** Source of origin of the 88 *L. monocytogenes* serogroup IVb isolates from Europe associated with the CC cluster.

Clonal Complex	Source of Origin					Tot
	Food	Animal	Human	Environment	N.R.^1^	
CC1	3 (11%)	4 (14%)	19 (68%)	1 (4%)	1 (4%)	28
CC2	5 (26%)	1 (5%)	11 (58%)	0	2 (11%)	19
CC4	1 (25%)	0	2 (50%)	0	1 (25%)	4
CC6	2 (22%)	2 (10%)	4 (44%)	1 (11%)	0	9
CC54	0	0	4 (100%)	0	0	4
CC315	2 (22%)	1 (11%)	5 (56%)	1 (11%)	0	9
CC388	0	15 (100%)	0	0	0	15

^1^ NR: not reported.

## Data Availability

This Whole Genome Shotgun project has been deposited at DDBJ/ENA/GenBank under the accession JAHALW000000000. The version described in this paper is version JAHALW010000000.
